# Current challenges in the endovascular treatment of medium vessel occlusions

**DOI:** 10.3389/fstro.2023.1242961

**Published:** 2023-08-15

**Authors:** Aaron Rodriguez-Calienes, Juan Vivanco-Suarez, Mahmoud Dibas, Daniel Casanova, Milagros Galecio-Castillo, Mudassir Farooqui, Santiago Ortega-Gutierrez

**Affiliations:** ^1^Department of Neurology, University of Iowa Hospitals and Clinics, Iowa City, IA, United States; ^2^Neuroscience, Clinical Effectiveness and Public Health Research Group, Universidad Científica del Sur, Lima, Peru; ^3^Facultad de Medicina, Universidad de Valparaíso, Campus San Felipe, San Felipe, Chile; ^4^Laboratorio de Neuroanatomía Microquirúrgica (LaNeMic) Facultad de Medicina, Universidad de Buenos Aires, Buenos Aires, Argentina; ^5^Department of Neurology, Neurosurgery and Radiology, University of Iowa Hospitals and Clinics, Iowa City, IA, United States

**Keywords:** stroke, endovascular, thrombectomy, medium vessel occlusion, MeVO

## Abstract

Medium vessel occlusions (MeVOs) account for 25%−40% of acute ischemic stroke (AIS). While mechanical thrombectomy is the standard-of-care for selected patients with large vessel occlusion (LVO), there is currently a lack of level I evidence of the safety and efficacy of endovascular treatment (EVT) for MeVOs. Several randomized clinical trials (RCTs) have attempted to answer this relevant clinical question. However, several questions related to the EVT of MeVO stroke may remain unanswered even after successful completion of these trials: What is the optimal EVT approach for secondary MeVOs? Is EVT beneficial for posterior circulation MeVOs? Is pre-EVT intravenous thrombolysis better than EVT alone? What is the optimal first line thrombectomy technique for these lesions? Are the outcome assessment tools used for LVOs appropriate for MeVOs? Upcoming evidence and the natural evolution and development of new technologies will aid in overcoming these challenges.

## 1. Introduction

Medium vessel occlusions (MeVOs) account for 25% to 40% of acute ischemic stroke (AIS) (Saver et al., 2020). While mechanical thrombectomy is the standard-of-care for selected patients with large vessel occlusion (LVO) (Powers et al., 2019), there is currently no high-level evidence of the safety and efficacy of endovascular treatment (EVT) for MeVOs. Recently, several meta-analyses using nonrandomized data have assessed the benefit of EVT in MeVOs, suggesting promising safety and efficacy (Barchetti et al., 2020; Waqas et al., 2021; Bilgin et al., 2022; Loh et al., 2022, 2023; Rodriguez-Calienes et al., 2023; Toh et al., 2023). Nevertheless, despite the available evidence, some questions related to the EVT of MeVO stroke remain unanswered. Overcoming these challenges during or after conclusion of the ongoing randomized control trials (RCTs) on MeVO is of relevance given that EVT for MeVOs could be a promising next step forward in AIS treatment (Goyal et al., 2020).

## 2. MeVO definitions

**Figure 1:**
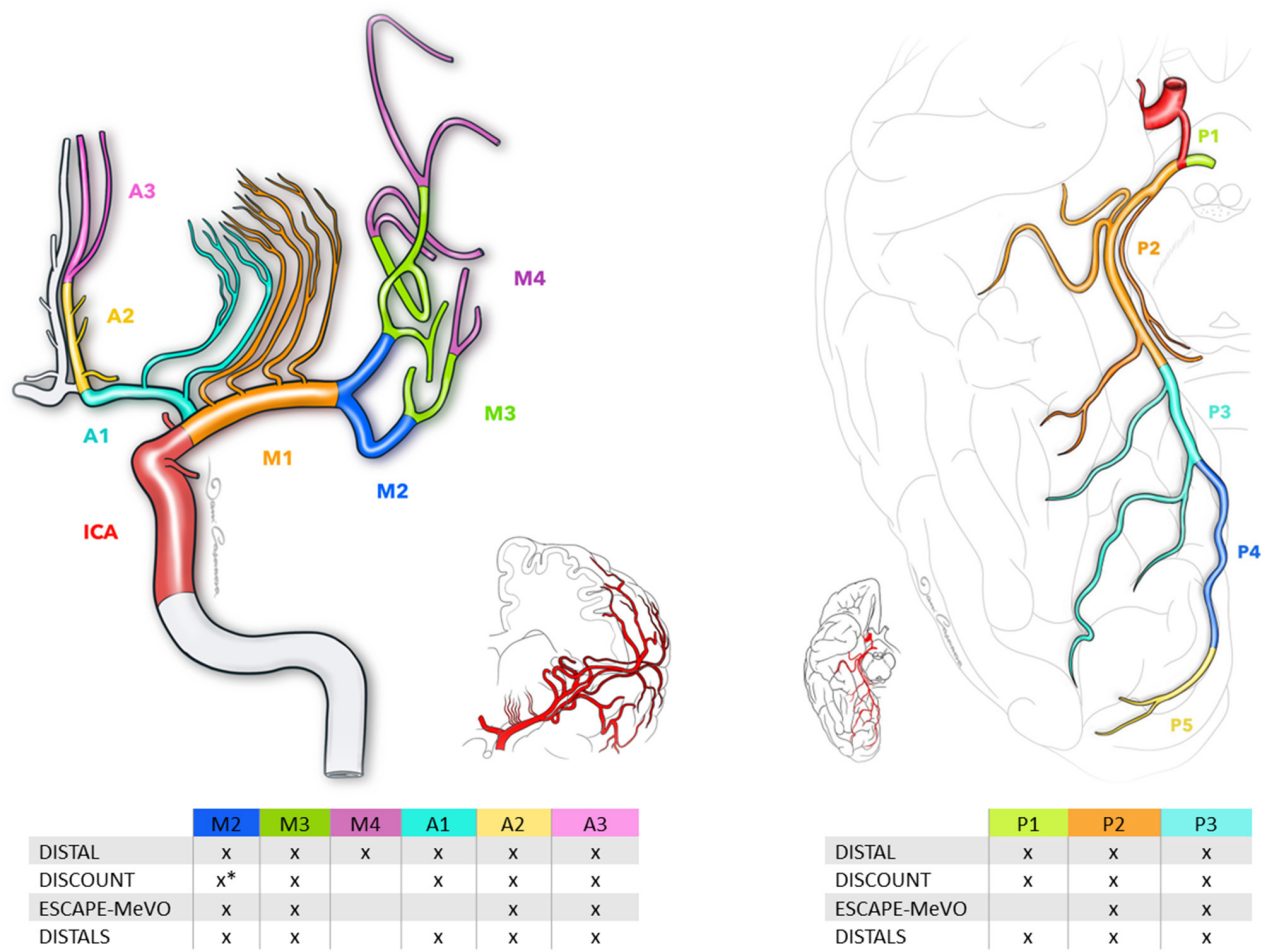
Schematic of medium vessel occlusions definitions, as defined by current randomized trials. The middle cerebral artery comprises three segments: M2, M3, and M4. The anterior cerebral artery consists of segments A1, A2, and A3, while the posterior cerebral artery includes segments P1, P2, and P3. ^*^Distal portion of the M2 segment of the middle cerebral artery.

There are different definitions for MeVOs in the literature. Among the ongoing RCTs, there are some differences between the trials regarding the definition of MeVOs (Figure 1). For example, the EnDovascular Therapy Plus Best Medical Treatment (BMT) vs. BMT Alone for MedIum VeSsel Occlusion sTroke (DISTAL) trial defines them as an occlusion of the co-/non-dominant M2, the M3/M4 segment of the middle cerebral artery (MCA), the A1/A2/A3 segment of the anterior cerebral artery (ACA) or the P1/P2/P3 segment of the posterior cerebral artery (PCA). The Evaluation of Mechanical Thrombectomy in Acute Ischemic Stroke Related to a Distal Arterial Occlusion (DISCOUNT) trial identifies MeVOs as an occlusion in one the following: distal M2 mainly above the mid-height of the insula, M3 segment, the A1/A2/A3 segment of the ACA or the P1/P2/P3 segment of the PCA. On the other hand, the EndovaSCular TreAtment to imProve outcomEs for Medium Vessel Occlusions (ESCAPE-MeVO) trial defines a MeVO as an occlusion in M2, M3 segment, A2, A3, P2 or P3 segment, while the Distal Ischemic Stroke Treatment With Adjustable Low-profile Stentriever (DISTALS) trial defines it as an occlusion within the territory of the ACA segments, a non-dominant or co-dominant M2 MCA segment, an M3 MCA, or the PCA segments. The definitions, inclusion criteria and primary endpoints of MeVOs are summarized in Table 1.

**Table 1:** Summary of ongoing MeVO trials including MeVO definitions, inclusion criteria, and primary study outcomes.

**Trial**	**Country**	**MeVO definition**			**Clinical inclusion criteria**	**Primary outcome**
		**MCA**	**ACA**	**PCA**		
DISTAL (NCT05029414)	Switzerland	Co-/non-dominant M2, M3, M4	A1, A2, A3	P1, P2, P3	NIHSS of ≥ 4 or symptoms deemed clearly disabling by treating physician (i.e. aphasia, hemianopia, etc.)	mRS at 90 days
DISCOUNT (NCT05030142)	France	Distal M2^*^, M3	A1, A2, A3	P1, P2, P3	NIHSS ≥ 5	mRS 0-2 at 3 months
ESCAPE-MeVO (NCT05151172)	Canada	M2, M3	A2, A3	P2, P3	NIHSS ≥ 5, or NIHSS 3-5 with disabling deficit (e.g. hemianopia, aphasia, loss of hand function)	mRS at 90 days
DISTALS (NCT05152524)	USA	Non-dominant or co-dominant M2, M3	ACA	PCA	NIHSS 4-24, or NIHSS 2-24 for patients with aphasia and/or hemianopia	Successful reperfusion without sICH^**^

MeVO, Medium vessel occlusion; MCA, Middle cerebral artery; ACA, Anterior cerebral artery; PCA, Posterior cerebral artery; USA, United States of America; NIHSS, National Institute of Health Stroke Scale; mRS, Modified Rankin scale; sICH, Symptomatic intracranial hemorrhage.

* Above the mid-height of the insula.

** Successful reperfusion is defined as >50% reduction in substantial hypoperfusion (Tmax >4 s) volume between baseline and 24 ± 6 h of randomization. sICH is defined as any parenchymal hematoma type 2, remote intracerebral hemorrhage, subarachnoid hemorrhage, or intraventricular hemorrhage that is the predominant cause of ≥4-point NIHSS deterioration at 24 ± 6 h of randomization.

## 3. Primary and secondary medium vessel occlusions

MeVOs are not all identical and can be classified based on the underlying mechanism by which they occur (Goyal et al., 2020). Primary MeVOs arise “*de novo*” with underlying mechanisms very similar to LVOs. On the other hand, secondary MeVOs arise from LVOs mainly due to EVT-induced clot fragmentation or spontaneous clot migration. Secondary MeVOs that originate from more proximal occlusions can result in a larger infarct area and are associated with a worse 24-h Alberta Stroke Program Early Computed Tomography Score due to the ischemic infarct growth caused by the initial LVO (Goyal et al., 2021). Therefore, they are expected to initially present with more severe clinical presentations and with more neurological deficits (Goyal et al., 2021). Moreover, there is some evidence to support greater clot fragility in secondary spontaneous EVT MeVOs. Thus, it would be more challenging to treat secondary non-EVT MeVOs in comparison to primary MeVOs, given the increased risk of thrombus fragmentation following EVT (Goyal et al., 2021). On the contrary, EVT-related MeVOs are more often treated than primary MeVOs.

A recent systematic review and meta-analysis showed that EVT for primary and secondary MeVOs is efficient and safe (Rodriguez-Calienes et al., 2023). The majority of the available studies focused on primary MeVOs with a primary-to-secondary MeVO ratio of 3.3:1 (Rodriguez-Calienes et al., 2023). The reason is that it is challenging to enroll patients who experience secondary MeVOs, particularly those that are diagnosed during digital subtraction angiography after initial suspicion of LVO in non-invasive imaging, given the constraints of consent for randomizing a patient during an ongoing procedure (Rodriguez-Calienes et al., 2023). In addition, identifying secondary MeVOs that are not EVT-induced due to spontaneous or systemic thrombolysis may only be done following the repetition of vascular imaging prior to EVT, which is not practical and causes important treatment delays. The main features of the effects of EVT in secondary MeVOs are summarized in Table 2.

**Table 2:** Summary of studies on secondary medium vessel occlusion strokes treated with endovascular thrombectomy.

**References**	**Study design**	**N**	**Age (years)**	**NIHSS**	**Onset-to-groin time (hours)**	**Location**	**MT Technique**	**mTICI 2b-3 (%)**
Onal et al. (2020)	R, SC	9	-	13 [10.3–15]	-	M3:3; A3:4; P3:1	SR	100
Pfaff et al. (2016)	R, SC	30	64 ± 13	18 (13–23)	3.5 [2.3–4.8]	A2:7; A3:16; A4-A5:7	SR	73
Grossberg et al. (2018)	R, SC	13	61.5 ± 19.1	17 [12–22]	6.9 ± 5.1	M3:13	SR, DA	53.8
Rikhtegar et al. (2021)	R, SC	71	74 ± 15	11 [0–31]	-	M3:48; M4:6; A3:9; P3:2; SCA:5; PICA:1	SR	80.3
Altenbernd et al. (2018a)	R, SC	27	69 ± 11.4	15 [12–18]	1.8 [1.6–2]	dM2-M3:27	DA	100
Settecase (2019)	R, SC	13	-	18.5 [12–25]	2 ± 0.6	dM2:5; M3:4; M4:1; A4:3	DA	100
Haussen et al. (2016)	R, SC	8	51 ± 20	19 ± 5	8.8 ± 4.8	M3:5; A5:1; P2-P3:1; P3:1	SR	75
Ozdemir et al. (2022)	R, MC	13	67.6 ± 12.7	18 [17–23]	5.6 [4.5–6.4]	M3:13	SR, IA	53.8
Grieb et al. (2022)	R, SC	13	71.6 (35–89)	14	0.8 ± 0.1	M3: 11; M4: 2	DA	61.5
Styczen et al. (2021)	R, MC	11	-	14 [9–21]	2.5 [1.5–3.9]	SCA:8; AICA:2; PICA:1	SR, DA	81.8
Crockett et al. (2019)	R, SC	11	-	18 [12–20]	-	dM2: 2; M3:3; A2:1; A3:2; P2:1; P3:1; SCA:1	DA	81.8
Miszczuk et al. (2021)	R, MC	18	-	-	-	P2:11; P3:7	SR, DA	83.3
Fischer et al. (2022)	R, MC	13	-	13	-	M3:6; A2:5; A3:1; A4:1	SR	92.3
Miszczuk et al. (2021)	R, SC	41	73 [62–82]	17 [15–21]	-	A2:19; A3:4; A5:18	SR, DA	83

N, number of participants; R, Retrospective; P, Prospective; SC, Single center; MC, Multicentric; NIHSS, National Institutes of Health Stroke Scale; MT, Mechanical thrombectomy; ASPECTS, Alberta Stroke Program Early Computed Tomography Score; DMVO, Distal medium-vessel occlusion; IVT, Intravenous thrombolysis; mTICI, Modified thrombolysis in cerebral infarction; SR, Stent-retriever; DA, Direct aspiration; dM2, Distal M2; SCA, Superior cerebellar artery; AICA, Anteroinferior cerebellar artery; PICA, Posteroinferior cerebellar artery.

## 4. Presentation of posterior circulation occlusions

The presentation of isolated posterior circulation MeVOs may be vague, leading to late admission and consequent ineligibility to thrombolytic treatment, especially in patients with mild deficits (Sommer et al., 2017). Of note, some of the important eloquent brain regions, including the primary visual cortex and the thalami, can be affected by the lack of blood supply due to posterior circulation MeVOs, which can result in severe and detrimental effects on quality of life (Schmahmann, 2003; Ryan et al., 2010; Sand et al., 2016). Also, the use of the National Institutes of Health Stroke Scale (NIHSS) score for posterior circulation MeVOs is not representative and can result in lower NIHSS cutoff values as compared to anterior circulation strokes (Sato et al., 2008). As a result, the quest for more efficacious and safe clinical evaluation modalities for posterior circulation MeVOs is still ongoing.

Unlike anterior circulation MeVOs, there is limited evidence related to the efficacy of EVT for posterior circulation MeVOs. Moreover, EVT is usually avoided in patients who present with mild deficits and are eligible for thrombolytic treatment (Seners et al., 2020). A multicenter case-control study by Meyer et al. revealed promising results with successful revascularization rates of 87.4% and symptomatic intracranial hemorrhage (sICH) rates of 4% (Meyer et al., 2021). In addition, a recent meta-analysis performed by our group that included distal MeVOs (DMVOs) in the P2-P5 vascular territory showed high rates of successful revascularization (81%) and lower sICH rates (3%) (Rodriguez-Calienes et al., 2023). Although these results are encouraging, they are suggestive rather than conclusive, and there is still a need for larger RCTs for further investigation.

## 5. Thrombectomy with or without intravenous thrombolysis

The role of intravenous thrombolysis (IVT) in LVOs in patients eligible for mechanical thrombectomy is a subject of debate. Theoretically, adding IVT may contribute to achieving early reperfusion of the ischemic territory before EVT (Desilles et al., 2015; Seners et al., 2016; Tsivgoulis et al., 2018; Ospel et al., 2021), increasing reperfusion rates with fewer recanalization attempts (Fischer et al., 2018), and may improve outcomes in patients with failed thrombectomy reperfusion attempts (Rozes et al., 2022). However, the theoretical risk of distal clot embolization (Ohara et al., 2021) and intracranial hemorrhage, the potential delays for arterial puncture, and the elevated cost are considerable disadvantages (Fischer et al., 2017; Ospel et al., 2022).

Regarding MeVOs, a multinational survey showed that more than 50% of physicians would perform EVT alone in M3, A2, and P2 occlusions if the patient is ineligible for IVT; however, if the patient is eligible for IVT, 40% would offer EVT in the A2 and P2 scenarios but only 18% would offer EVT in the M3 scenario (Kappelhof et al., 2022). This reflects that the willingness to use IVT in combination with EVT for MeVOs is low. Interestingly, pharmacologic fibrinolysis is more effective for the smaller clot burden of MeVOs than the large clot burdens of LVOs (Kim et al., 2015; Yoo et al., 2018); however, IVT alone recanalizes only one-third to one-half of visualized thrombi (Saver et al., 2020). In addition, since IVT may impact the risk of intraprocedural clot fragmentation, it is possible that the fear of causing an IVT-induced secondary MeVO may be considered as a reason to withhold IVT before EVT (Goyal et al., 2021). Moreover, the subtle and diverse clinical syndromes observed with MeVO stroke may contribute to delayed presentation times, which make these patients ineligible for IVT (Saver et al., 2020).

To date, no comparative analysis between EVT alone vs. IVT with EVT in MeVOs has been reported but some retrospective cohorts suggest that IVT before EVT can achieve high reperfusion rates with no risk of hemorrhage. In the study by Altenbernd et al., pre-EVT IVT was used in 91.4% of M2 and M3 occlusions. Successful reperfusion (modified thrombolysis in cerebral infarction [mTICI] 2b-3) was observed in 100% and complete reperfusion (mTICI 3) in 82.8%, while only 3.4% presented sICH (Altenbernd et al., 2018b). Similarly, in the study by Castro-Afonso et al., successful reperfusion (mTICI 2b-3) was observed in 89% of M2 occlusions treated with IVT before EVT (De Castro Afonso et al., 2019). In addition, Styczen et al. reported a successful reperfusion rate of 90% in a cohort of posterior inferior cerebellar artery, anterior inferior cerebellar artery, and superior cerebellar artery occlusions treated with IVT before EVT (Styczen et al., 2021).

## 6. Combined vs. single-device techniques

The recent introduction of new generation small caliber catheters and low-profile stent retrievers (SR) has allowed access to the sites of distal occlusions (Saver et al., 2020); however, the optimal specific endovascular technique for MeVOs remains unknown. A few years ago, the use of a primary combined approach (SR with direct aspiration [DA]) and advancing the system in a tri-axial manner was troublesome due the insufficient catheter length and diameter discrepancies (Ospel and Goyal, 2021). Thus, single-device thrombectomy approaches (SR or DA) were traditionally used for this subgroup of patients. The meta-analysis of DMVOs performed by our group found higher rates of successful reperfusion (mTICI 2b-3) and favorable functional outcomes with DA techniques compared to SR techniques (Rodriguez-Calienes et al., 2023). In addition, the pooled rates of sICH and 90-day mortality were lower in the DA group (Rodriguez-Calienes et al., 2023). On the other hand, the meta-analysis of proximal and DMVOs by Loh et al. found higher odds of functional independence and lower odds of mortality in the SR/primary combined group compared to DA alone group, while reperfusion and sICH rates were similar between the two groups (Toh et al., 2023). Nevertheless, when they compared SR alone with DA alone, there were no differences in the odds of functional independence, sICH, or mortality (Toh et al., 2023). Therefore, we can infer that for certain outcomes the superior effect observed in the SR/primary combined group compared to the DA group may be primarily influenced by the subpopulation of the combined approach.

Compared to single-device approaches, combined techniques can ensure the capture of thromboembolic clots from both sides via a SR inserted distally and an aspiration catheter placed proximally, thus enhancing clot removal (Massari et al., 2016; McTaggart et al., 2017; Maus et al., 2018). In addition, the “pinning technique” (deployment of a SR through an intermediate catheter engaging the clot while exerting local aspiration) minimizes deformation of the tortuous distal vessels (Yoo and Andersson, 2017). Thus, a combined approach can provide advantages in the treatment of MeVOs by minimizing the risk of distal clot embolization and device-withdrawal risks for subarachnoid hemorrhage caused by small vessel size and tortuosity (Haussen et al., 2020; Pérez-Garciá et al., 2020).

Recently, several studies have suggested the superiority of the combined approach vs. the single-technique approach. The meta-analysis of DMVOs by Loh et al. compared first-line combined techniques with single-device techniques and found higher odds of reperfusion at first pass and lower odds of sICH with the combined approach, but no differences in final reperfusion, functional independence, or mortality (Loh et al., 2023). Similarly, the systematic review by Biling et al. described higher rates of successful reperfusion and functional independence with a combined treatment technique compared to DA or SR alone (Bilgin et al., 2022). Finally, despite the limitations of the current generation of thrombectomy devices in treating MeVOs, advances in technology and techniques may result in new tools specifically suited for MeVOs, which will allow the identification of an optimal endovascular technique to achieve better outcomes.

## 7. Outcomes assessment

Given that the occlusion location is more distal in MeVOs and that these MeVOs usually result in a smaller ischemic area, the outcomes would be expected to be better in MeVOs as compared to LVO strokes (Ospel and Goyal, 2021). Nonetheless, the results of the INTERRSeCT and PRove-IT trials revealed that almost one out of four MeVO patients do not attain functional independence with the standard treatment. Additionally, only half of the patients with MeVOs end up with an excellent outcome (Ospel et al., 2020). Therefore, the use of EVT in MeVOs might be plausible. The use of EVT has gained a lot of attention lately and was shown to be associated with decent efficacy and safety (Rodriguez-Calienes et al., 2023). The evidence is still in its infancy though, given the lack of randomized trials to prove this. To better evaluate the efficacy of EVT for MeVOs, especially since the outcomes of MeVOs are expected to be milder than LVO, it is more justified to use more restrictive outcomes and outcomes that are tailored to MeVOs. Instead of using good outcomes (i.e. modified Rankin scale [mRS 0-2]), which were adopted by the majority of the studies in the literature, it appears more reasonable to opt for excellent outcomes (i.e. mRS 0-1) or shift analysis (Ospel and Goyal, 2021). Despite that, the mRS and NIHSS are still not fully representative of MeVOs and, thus, do not grasp the whole clinical picture of the patient. Clinical deficits including isolated abulia, alexia and agraphia are not caught in these prior scorings, which really questions the suitability of these scoring systems for MeVOs, and calls for studies to develop outcomes that are tailored to MeVOs (Ospel and Goyal, 2021). Similarly, angiographic outcomes using the TICI score are not deemed reflective for MeVOs as the majority of patients have TICI 2b at baseline (Ospel and Goyal, 2021). As a result, there is a need to develop a comprehensive angiographic scoring system for MeVOs.

## 8. Future directions

Although the clinical syndromes associated with MeVOs are heterogenous and fractionated, the natural history of the ischemic lesions they cause is poor and frequently disabling. The safety and efficacy profile of EVT is favored by the continual evolution and development of imaging technologies, devices, and techniques. However, the development and acceptance of new outcome measurement tools are needed to objectively quantify the safety and efficacy of performing EVT in these lesions. Currently, there are 4 ongoing RCTs on primary MeVO (Table 1): (1) NCT05029414, DISTAL is a multicenter, parallel assignment, open-label, superiority trial based in Europe that expects to enroll 526 patients by December 2024, and in which all EVT techniques are allowed; (2) NCT05030142, DISCOUNT is a multicenter, parallel assignment, open-label trial based in France that aims to enroll 488 participants by February 2024; EVT will be performed with a specific selection of SRs; (3) NCT05151172 ESCAPE-MeVO is a multicenter, open-label trail based in Canada that aims to enroll 530 participants by December 2025; all the first attempts of EVT will be performed with the Solitaire X (Medtronic, USA) SR; and (4) NCT05152524, DISTALS is an international (United States and Europe) multicenter, open-label trial that aims to enroll 168 participants by January 2024, and in which all EVTs must be performed with Tigertriever 13 (Rapid Medical, Yoqneam, Israel).

While we await the results of the current ongoing clinical trials, several interventionalists are already routinely treating primary and secondary MeVOs. Hopefully, the randomized results provide additional evidence to standardize the best selection of imaging protocols, treatment indication criteria, and techniques that favor the best clinical outcomes.

## Author contributions

AR-C, JV-S, and MD wrote the sections of the manuscript. DC designed the illustrations. MG-C and MF contributed to manuscript revision. SO-G contributed to concept and design of study and approved the submitted version. All authors contributed to the article and approved the submitted version.
